# Trojan-Like Internalization of Anatase Titanium Dioxide Nanoparticles by Human Osteoblast Cells

**DOI:** 10.1038/srep23615

**Published:** 2016-03-29

**Authors:** A. R. Ribeiro, S. Gemini-Piperni, R. Travassos, L. Lemgruber, R. C. Silva, A. L. Rossi, M. Farina, K. Anselme, T. Shokuhfar, R. Shahbazian-Yassar, R. Borojevic, L. A. Rocha, J. Werckmann, J. M. Granjeiro

**Affiliations:** 1 Directory of Life Sciences Applied Metrology, National Institute of Metrology Quality and Technology, Rio de Janeiro, Brazil; 2 Brazilian Branch of Institute of Biomaterials, Tribocorrosion and Nanomedicine (IBTN), University Estadual Paulista, Faculty of Sciences, Bauru, São Paulo, Brazil; 3 Postgraduate Program in Translational Biomedicine, University of Grande Rio, Duque de Caxias, Brazil; 4 Welcome Trust Centre for Molecular Parasitology, University of Glasgow, United Kingdom; 5 Brazilian Center for Research in Physics–Rio de Janeiro, Brazil; 6 Biomineralization laboratory, Institute of Biomedical Sciences, University Federal do Rio de Janeiro, Rio de Janeiro, Brazil; 7 Institut de Science des Materiaux de Mulhouse–CNRS UMR7391, Universite de Haute-Alsace, Mulhouse, France; 8 Department of Bioengineering, University of Illinois at Chicago, Chicago 60607, United States; 9 Department of Mechanical and Industrial Engineering, University of Illinois at Chicago, 60607, United States; 10 Center of Regenerative Medicine, Faculty of Medicine–FASE, Petrópolis, Brasil; 11 Physics Department, University Estadual Paulista, Bauru, São Paulo, Brazil; 12 Dental School, Fluminense Federal University, Niterói, Brazil

## Abstract

Dentistry and orthopedics are undergoing a revolution in order to provide more reliable, comfortable and long-lasting implants to patients. Titanium (Ti) and titanium alloys have been used in dental implants and total hip arthroplasty due to their excellent biocompatibility. However, Ti-based implants in human body suffer surface degradation (corrosion and wear) resulting in the release of metallic ions and solid wear debris (mainly titanium dioxide) leading to peri-implant inflammatory reactions. Unfortunately, our current understanding of the biological interactions with titanium dioxide nanoparticles is still very limited. Taking this into consideration, this study focuses on the internalization of titanium dioxide nanoparticles on primary bone cells, exploring the events occurring at the nano-bio interface. For the first time, we report the selective binding of calcium (Ca), phosphorous (P) and proteins from cell culture medium to anatase nanoparticles that are extremely important for nanoparticle internalization and bone cells survival. In the intricate biological environment, anatase nanoparticles form bio-complexes (mixture of proteins and ions) which act as a kind of ‘Trojan-horse’ internalization by cells. Furthermore, anatase nanoparticles-induced modifications on cell behavior (viability and internalization) could be understand in detail. The results presented in this report can inspire new strategies for the use of titanium dioxide nanoparticles in several regeneration therapies.

Titanium and Ti-based alloys are the most widely used metallic materials for dental and orthopedic implants^1^. However, a significant amount of prostheses and dental implant failures are associated with surface degradation phenomena, by the combined action of corrosion and wear (tribocorrosion) that generate metallic ions and wear debris^2^,^3^,^4^. Clinical studies have already demonstrated that titanium dioxide particles together with metallic ions released from implants accumulate in peri-implant tissues. Particles size range from nanometer to micrometer scale^4^,^5^,^6^,^7^,^8^,^9^. Nanoform debris can enter into the circulatory system, accumulating in lungs, liver, spleen and bone marrow, and contributes to the development of nanoparticle-associated diseases in the respiratory or cardiovascular systems, which can lead even to carcinomas^3^,^5^. There is growing evidence that titanium dioxide nanoparticles directly affect bone cells viability, proliferation, differentiation and mineralization^6^,^8^,^9^,^10^,^11^,^12^,^13^,^14^,^15^. Recent reports indicate that titanium dioxide particles activate the innate immune system leading to periprosthetic bone resorption (osteolysis) with subsequent implant failure and the need of an additional surgery for the patient^4^,^7^.

Interaction between titanium implants and adjacent tissues and cells occurs and has to be studied in two contexts. The first one deals with interaction of implant with bone cell progenitors and bone-derived cells of the adjacent tissues. They have to adhere and spread on the implant, in order to deposit a new bone onto the implant surface and promote the stable mechanical and biological integration of the structures. The compatibility of the implant surface with bone cells has been the subject of extensive studies, and both physical and chemical properties of the titanium implant surface were shown to be relevant for the optimal cell-implant integration. The progressive increase of adhesive cell contacts with solid substrates induces cell spreading, until they cover all the available surface and form a continuous cell layer^16^,^17^,^18^,^19^.

The second context deals with micro- and nano-particles, released from implants into the adjacent tissues. They can interact with the adjacent and already adherent cells on the implant, but they may also be washed by tissue-fluids and reach distant bone cells. Local inflammation increases the flux of intercellular liquids in inflamed tissues, and facilitates the migration of implant-derived particles. Cells cannot spread on nanoparticles that are very small, but the tendency to increase the cell-titanium interphase leads to membrane invagination around nanoparticles, resulting in their internalization. Thereafter, nanoparticles can interact with internal cell organelles and structures, leading to functional modifications and to intracellular lesions. Several published studies deal with nanomaterials toxicity in direct interactions of adherent cells with implant solid surfaces. However, there is a gap in our knowledge regarding correlation between the toxicity of the nanomaterial-derived particles and the bio-interface of bone cells^5^,^20^,^21^.

In both contexts, initial cell membrane interactions with extracellular compounds involve negative charges of the membrane glycocalyx. The extracellular cations, mainly Ca^++^, are the major actors in this interaction, and chelation of free Ca ions in cell culture is impeditive for the initial cell adhesion. The corresponding charges on the extracellular substrate are required for cell membrane-substrate interaction, and their density is determinant for the initial quality of cell adhesion. Subsequently, stable recognition and interaction among cell membrane receptor proteins and extracellular ligands can be established, involving in general the Arg-Gly-Asp (RGD) sequence-containing proteins, such as fibronectin, and the cell membrane receptors, most frequently integrins. Available extracellular substrate-bound Ca obviously facilitates the initial phases of cell adhesion. Integrins can move within the membrane, associate in groups, and coordinate the actin cytoskeleton organization. Forming dynamic focal-adhesions, they can activate intercellular signaling through cascades initiated by focal-adhesion kinases (FAK), and modulate cell movement and activation on solid substrates, as well as receptor-mediated phagocytosis of the membrane-adhered particles^16^,^22^,^23^.

In a recent study, within the first context of cell-substrate interactions, our group has developed a new multifunctional calcium-rich surface for dental implants^24^. An outermost nanometer-thick amorphous layer rich in calcium (Ca) and phosphorus (P) improved cell viability and metabolism, resulting in increased osteoblastic activity. This was a result of increased cell adhesion, as shown by full formation of actin stress fibers anchored in focal adhesions. Their increase is reported to be directly proportional to the available surface ligand density, which is determinant for mobilization of RGD sequences and the corresponding binding of integrins^22^.

In the present study, we have addressed the question of cell interactions with nanoparticles that mimic tribocorrosion products. They range from ions to complex structures, and are in contact with intercellular biological fluids, as well as with intercellular protein complexes, extracellular matrix, and molecules of the adjacent cell membranes. Several nanomaterial/biological interfaces are hence established, and they can be studied *in vitro*^25^,^26^. Ca and P ions are the first components of the cell/nanomaterial interphase, followed by proteins. Cell responses are dependent of the protein layer (protein corona) adsorbed to nanoparticle surface upon exposure to biological environments^25^,^26^,^27^,^28^. This protein corona creates an initial nano-bio interface that undergoes dynamic alterations as particles traffics onto or into cells^29^. Depending on the physicochemical characteristics of the nanoparticles and of the biological environment, selective proteins will stay bound to the particle, and this protein layer is likely to be what cells “see” and sense^4^,^8^. Recently Xu *et al.* showed the formation of a bio-complex (ions and proteins) as a result of the selective attachment of cell culture ions and proteins to CuO and ZnO nanoparticles, demonstrating that the level of complexity at this biointerface is higher^8^.

We employed titanium dioxide nanoparticles (anatase mineral phase) as a model to explore nano-bio interactions in biological environment, including nanoparticle internalization by cells. The objective was to correlate the events occurring during those interactions with toxicity induced in bone cells. High-resolution electron microscopy techniques combined with proteomics was employed to demonstrate that the bio-complexes formed outcomes in a kind of Trojan horse that enhances nanoparticle internalization by bone cells. Although the conditions tested here were not within the range of overt toxic conditions, cell cycle analyses suggested DNA damage for higher exposure conditions. Given the growing use of TiO_2_ nanoparticles, these findings raise concern about potential human health hazards.

## Results

**Figure 1:**
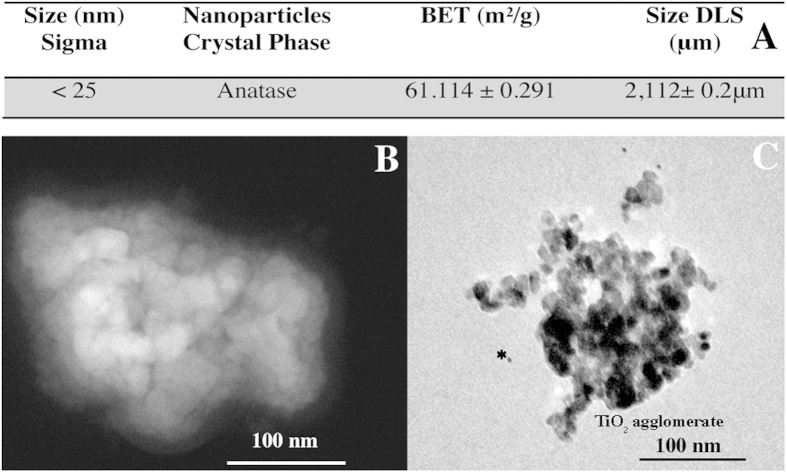
Physicochemical characterization of anatase nanoparticles: (**A**) primary particle size, XRD phase identification, surface area (BET), and size of titanium dioxide nanoparticles in water (DSL), (**B**) STEM image of anatase agglomerates in culture medium (**C**) Immunogold anti-albumin (*indicate small gold dots) demonstrating BSA adsorption on TiO_2_ aggregates observed by TEM.

Physiochemical characterization of anatase nanoparticles indicated that their specific surface area was ~61.14 m^2^/g (see table in Fig. 1A). Individual particles of approximately 25 nm were recognizable (Fig. 1B,C). An optimized dispersion protocol allowed the de-aggregation of anatase nanoparticles suspension that, in culture medium, presented a mean hydrodynamic size of 142.1 ± 5.6 nm (obtained by dynamic light scattering DLS), which is a similar size observed by Scanning Transmission Electron Microscopy (STEM). High-resolution TEM images demonstrated that albumin was surrounding anatase aggregates (see immunogold localization Fig. 1C) since albumin was used as a stabilizing agent.

**Figure 2:**
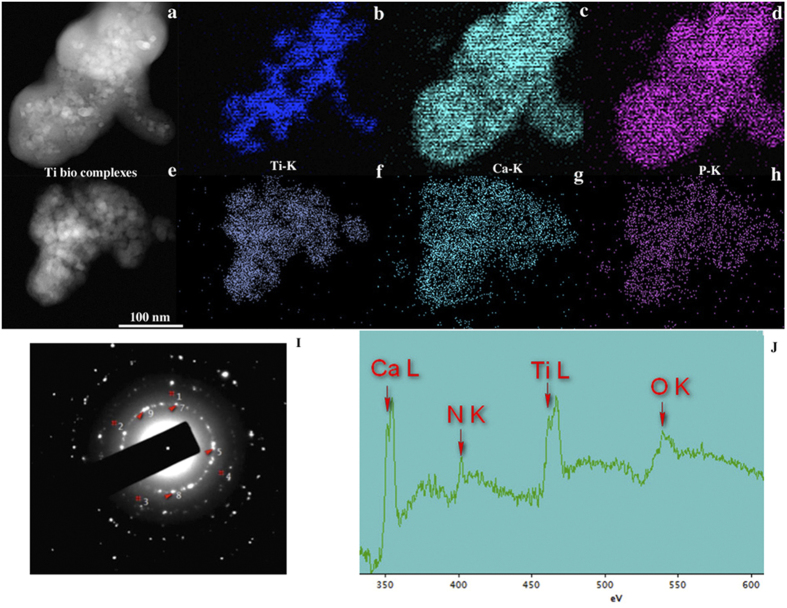
Concentration dependent of anatase bio-complexes formation in medium culture: 10 μg/mL anatase (**a**) Dark-field STEM image showing where the corresponding elemental maps were obtained; (**b**) STEM/EDS Ti-K map; (**c**) STEM/EDS Ca-K map; (**d**) STEM/EDS P-K map. 100 μg/mL anatase (**e**) Dark-field STEM image showing where the corresponding elemental maps were obtained; (**f**) STEM/EDS Ti-K map; (**g**) STEM/EDS Ca-K map; (**h**) STEM/EDS P-K map. (**I**) Diffraction pattern of {211} hydroxyapatite nanocrystals planes (marked as^#^) and anatase nanocrystal planes used for calibrating, d {101} = 0.351 nm (5, 7,8 and 9). (**J**) EELSpectrum of 100 μg/mL of anatase in culture medium.

Figure 2 presents STEM elemental maps, electron diffraction pattern and electron energy loss spectrum (EELS) obtained from anatase nanoparticles (10 and 100 μg/mL) in culture medium. We observed that various elements from culture milieu, such as biological ions (Fig. 2C,D,G,H) and proteins (Fig. 2J) were bound to anatase nanoparticles forming a bio-complex. By the EDS elemental maps in STEM mode, it was possible to detected calcium (Ca) and phosphorous (P) deriving from the medium culture binding to anatase nanoparticles as well as some diffraction reflections corresponding to hydroxyapatite (HA). Furthermore, these bio-complexes were also formed when anatase nanoparticles were incubated in DMEM medium without fetal bovine serum and albumin stabilization (see Fig. S1 of complementary information). These results suggest that the formation of anatase bio-complexes is independent of specific proteins. The signal of nitrogen (N) (Fig. 2J), a component of proteins, was detected indicating the formation of a protein corona. After 24 h of anatase nanoparticles incubation with the complete cell culture medium, the protein and ion corona was also formed (data not shown).

**Figure 3:**
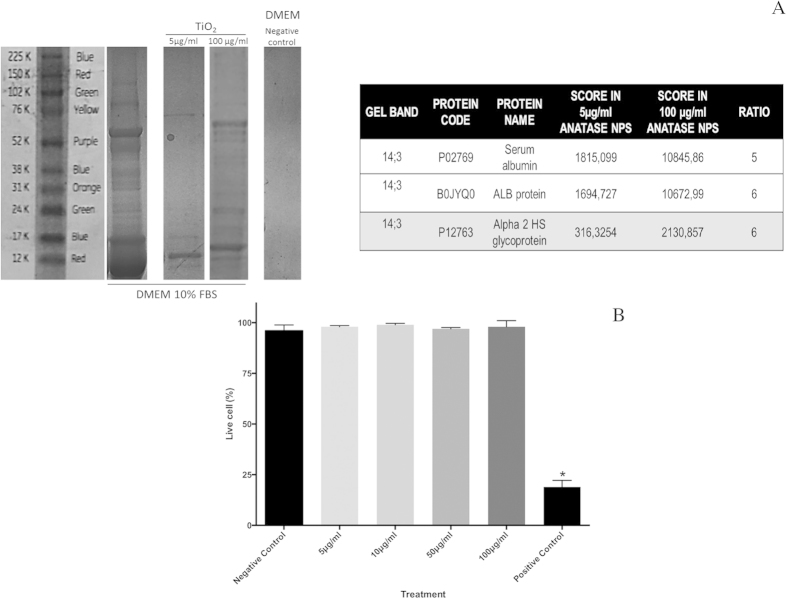
Osteoblasts viability upon anatase nanoparticles exposure: (**A**) Illustration of SDS-PAGE gels and identified bands. (**B**) Live and dead assay of osteoblast viability after 72 h of nanoparticle exposure.

The adsorption of proteins to anatase nanoparticles in serum-containing medium has been also investigated. SDS gel electrophoresis demonstrated that the protein adsorbed on anatase increased with the increasing of nanoparticles concentration (Fig. 3A), and that serum albumin as well as other glycoproteins (ALB protein and Alpha 2HS) were present. The anatase bio-complexes had no influence on osteoblasts viability after exposure for 72 hours. The percentage of dead cells was similar to the negative control (medium culture). The positive control (CuO) presented higher levels of death cells (Fig. 3B). No significant differences were observed on the population of necrotic and apoptotic cells exposed to the different concentrations of anatase nanoparticles (see anexin and PI apoptosis assay on Fig. S2A of complementary information). However, cell cycle analysis demonstrated arrest in G2/M phase for higher concentrations of anatase nanoparticles as well as an increased quantity of DNA fragments. This suggests a DNA damage and activation of DNA repair processes (Fig. 2B in complementary information).

**Figure 4:**
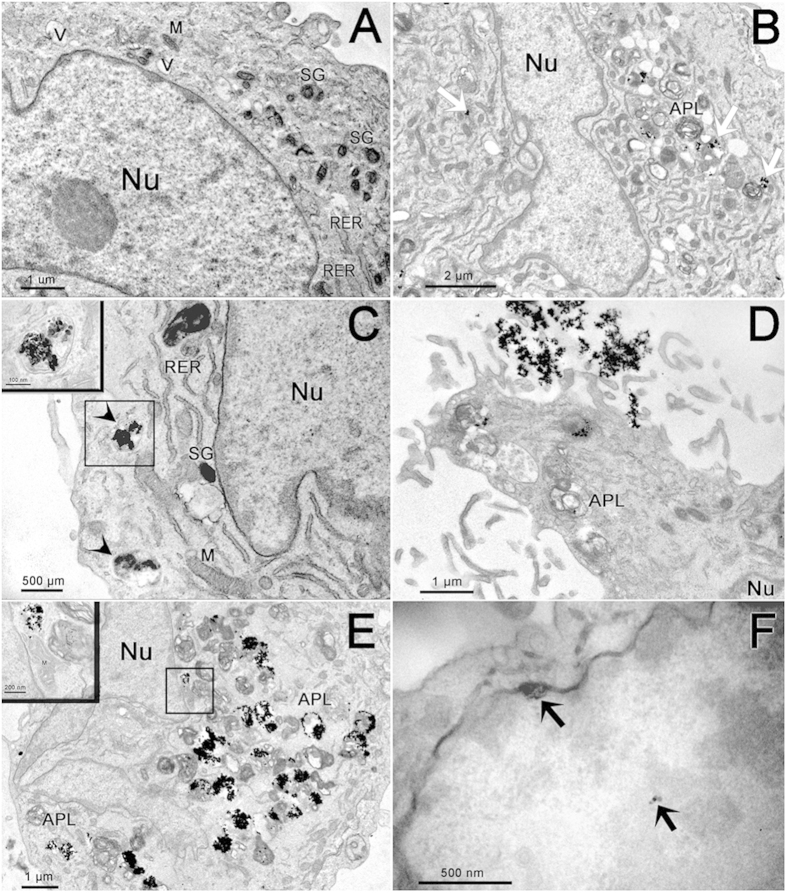
Electron micrographs of osteoblast cells: (**A**) Untreated cell. (**B–C**) Osteoblast cells treated with 5 μg/mL of anatase nanoparticles. Note the presence of nanoparticles distributed in cell cytoplasm (white arrows). In C we can observe anatase nanoparticles inside vesicles (arrow heads). Detail of these vesicles can be observed in the inset. (**D–F**) Osteoblast cells treated with 100 μg/mL of anatase nanoparticles. Note the large quantities of nanoparticles entering the cells (**D**) and internalized by cells (**E**). Inset shows nanoparticles next to cell nucleus and inside mitochondria. In (**F**) anatase nanoparticles inside the nucleus of the cell (black arrows). NPs–nanoparticles, APL–Autophagolysosomes, M–Mitochondria, Nu–Nucleus, RER–Rough Endoplasmic Reticulum, SG–Secretory Granules, V–Vesicles.

Although anatase bio-complexes did not show toxicity at the concentrations and times tested, they were internalized by osteoblasts, as observed in the TEM micrographs presented in Fig 4. As observed in Fig. 4A, the osteoblasts that were not in contact with anatase nanoparticles (untreated osteoblasts) presented morphology of typical secretory cells with round shape, irregular nucleus with dispersed chromatin and visible nucleoli, cytoplasm containing well-developed rough endoplasmic reticulum, large Golgi complex, electron dense mitochondria, vesicles and vacuoles containing fibrillar structures as well as secretory granules. When the cells were exposed to anatase nanoparticles in both concentrations, the nanoparticles were engulfed and internalized within the osteoblast cytoplasm as shown in Fig. 4 B–F. When osteoblasts were incubated with 5 μg/mL of anatase nanoparticles, they presented an ultrastructure similar to the untreated cells. However, some swollen mitochondria and some structures similar to autophagolysosomes could be observed in these cells, as shown in Fig. 4B. Anatase nanoparticles could also be seen in cell periphery, sometimes free in the cell cytoplasm (Fig. 4B) or associated with mitochondria, but mostly inside vesicles (Fig. 4C). Some cells presented features of necrotic lysis, such as advanced swelling of intracellular organelles associated with cell-membrane disruption and intensive vacuolization. Osteoblasts incubated with 100 μg/mL of anatase nanoparticles presented an ultrastructure and a nanoparticles internalization pattern similar to the former treatment, but with more evident vacuoles in cells cytoplasm, cell-membrane disruption and apparently bigger autophagolysossomes (Fig. 4D). Several cytoplasmic vesicles containing anatase nanoparticles were observed dispersed in cell cytoplasm and close to the cell nucleus (Fig. 4E). Also, nanoparticles associated to and inside mitochondria were observed (Fig. 4E). Several cells presented more than 50% of their cytoplasm occupied with anatase nanoparticles inside vesicles (Fig. 4E). Nanoparticles were also occasionally observed within the nucleus (Fig. 4F).

**Figure 5:**
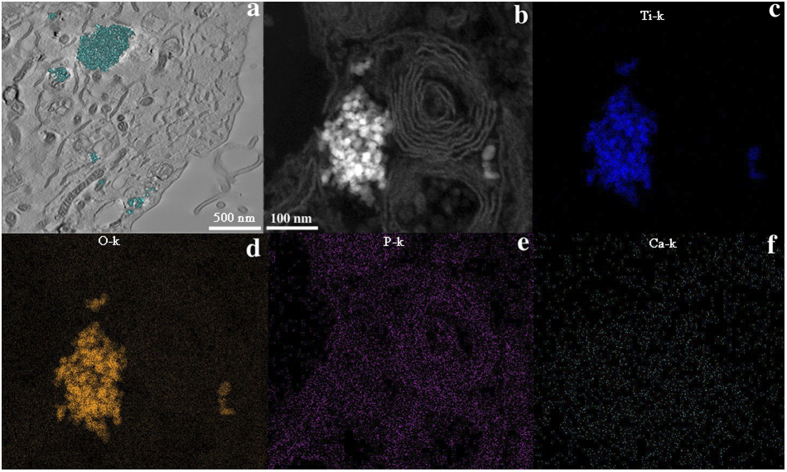
Anatase bio-complexes inside osteoblasts: (**a**) section obtained from a 3D tomographic reconstruction showing anatase internalization (5 μg/mL) in osteoblast cells; (**b**) high resolution Dark-field STEM image showing anatase internalized by an osteoblast from where the elemental maps were obtained; (**c**) STEM/EDS Ti-K map; (**d**) STEM/EDS O-K map; (**e**) STEM/EDS P-K map; (**f**) STEM/EDS Ca-K map.

The presented data suggest that anatase nanoparticles have not an overt toxicity to human osteoblasts causing cell death, even though they were internalized by cells. Morphological analysis of cell organelles however indicates cell suffering, and DNA analysis shows extensive fragmentation that may disturb gene expression and cause mutagenesis in cell replication. Electron tomography confirmed that most of the bio-complexes (in cyan color in the 3D model in Fig. 5A) were found inside vesicles in cell cytoplasm. No Ca was detected to be adsorbed to nanoparticles inside of the cell (Fig. 5F) while no conclusions can be obtained regarding phosphorous since its distribution is overlapping osmium (used as a post-fixative and as a marker for membranes) profile.

## Discussion

Wear debris and metal ion release from metallic biomaterials implanted into the human body is becoming a main cause of concern since they can cause adverse health effects^5^. Since Ti alloys are used in implants and prostheses that are in direct contact with bone cells, it is crucial to elucidate the interaction of Ti-based nanoparticles on primary human osteoblasts. Recent studies report that titanium dioxide nanoparticles are cytotoxic in different cell models, such as fibroblasts, macrophages, keratinocytes, and bronchiolar epithelial cell^30^. Until now, only a few studies have analyzed the effect of titanium dioxide nanoparticles and their possible cytotoxicity on human bone cells, using mostly murine cells or human osteosarcoma cell models^3^,^11^,^14^,^31^,^32^,^33^. Given the worldwide concerns about nanotoxicity, the potential cytotoxicity of anatase nanoparticles in bone cells and the influence of nano-bio interface on cellular internalization were studied in detail in the present work.

Several types of nanoparticles, quantum dot/hydroxyapatite composites, calcium phosphate nanoshells, and polymeric nanoparticles were demonstrated to be internalized by osteoblasts triggering cellular responses^34^. Recent studies report that titanium dioxide nanoparticles are internalized by mesenchymal stem/osteoprogenitor cells (MSC) causing cell death as well as a reduction in cell proliferation and differentiation. Also, it was suggested that osteogenic differentiation of MSC is dependent upon the size of nanoparticles^13^,^32^,^35^. Concerning to anatase nanoparticles effect on MC-3T3 cells, increased cell granularity and DNA fragmentation have been observed. In addition, those nanoparticles induced higher secretion of the pro-inflammatory cytokine (IL-6) known to mediate osteolysis^36^. Nonetheless, rutile debris seems to possess a lower bio reactivity on osteoblasts with a reduced release of inflammatory factors^37^. Exposure of human osteoblastic cells to titanium dioxide particles reduced their adhesion strength, migration and proliferation^15^.

It is important to consider that most of the studies involving titanium dioxide nanoparticles and bone cells did not take into account the ability of nanoparticles to interfere with *in vitro* toxicological assays, as well as to agglomerate when in contact with water as well as with biological fluids^38^. Invariably, working with titanium dioxide nanoparticles requires the optimization of a dispersion protocol in a biological matrix relevant to *in vitro* studies. In the present work, a protocol to disperse anatase nanoparticles in culture medium was developed and the nano-bio interface was explored. As it is known from the literature, when in contact with culture medium, a protein corona is formed around nanoparticles. This protein corona is strongly dependent on nanoparticle physicochemical characteristics, and it influences nanoparticle uptake and transport into the cell. Recently, Xu *et al.* demonstrated that the nano-bio interface surrounding nanoparticles is very complex. A bio-complex composed by a mixture of ions and proteins of the culture medium is selectively adsorbed at nanomaterials surface (as demonstrated for CuO and ZnO nanoparticles)^25^.

In the present work, the formation of a bio-complexes rich in Ca and P on titanium nanoparticles was observed, and some hydroxyapatite crystalline structures were also detected. In fact, Kokubo *et al.* have already reported surface bioactivity for bulk titanium covered by a titanium dioxide passive film^39^. Regarding anatase nanoparticles, the presence of hydroxyl groups possibly provides the site for calcium and phosphate nucleation^39^. The OH^−^ formed groups are negatively charged combining with the positively charged calcium ions (Ca^2+^) from medium culture through electrostatic forces. Subsequently, the negatively charged phosphate ions (PO_4_^3−^) combine with positively charged surface to form calcium phosphate. Because amorphous calcium phosphate is metastable, eventually it transforms into crystalline apatite.

**Figure 6:**
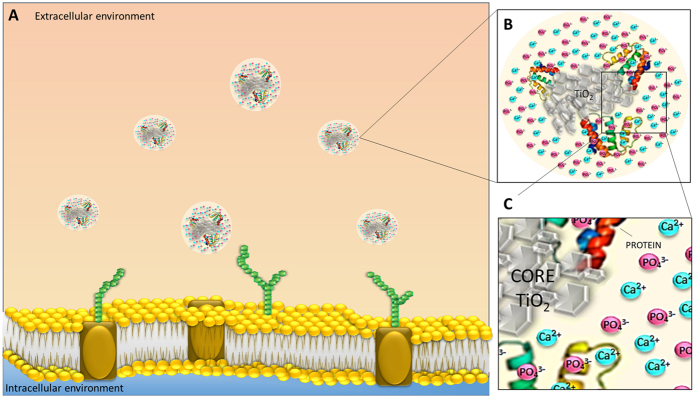
Anatase nanoparticles biointerface model at a cellular level: When anatase nanoparticles interact with biological milieu (**A**), they are instantaneously coated by proteins and by selective ions due to nanoparticle surface reactivity (**B,C**). This selective ion adsorption may be analogous to that seen in the formation of protein coronas, which is well known to be influenced by physical-chemical characteristics of nanoparticles. Calcium, phosphorus and biomolecules selectively adsorbed mask anatase nanoparticles facilitating nanoparticle internalization.

In the present study, it is suggested that what osteoblasts sense and contact with is a bio-complex, rich in calcium, phosphorous, hydroxyapatite and organic molecules (proteins). This bio-complex masks anatase nanoparticles during internalization (see model Fig. 6). Mass spectrometry identified serum albumin, ALB protein and Alpha 2 HS glycoprotein. Serum albumin is present in culture medium and we also used it as a stabilizing agent. The other glycoproteins detected on the surface of TiO_2_ are very important in transport. ALB protein has a good binding capacity for water, Ca^2+^, Na^+^, K^+^, fatty acids, hormones, bilirubin and drugs and also in endocytosis. Alpha 2 HS glycoprotein promotes endocytosis, possesses opsonic properties and influences the mineral phase of bone; it shows also affinity for calcium ions. All of them demonstrate a good binding affinity to calcium contributing to the enhancement of Ca content on the bio-complex, since the adsorption of Ca and P is independent of proteins (see Fig. 1 of complementary information). Our data on the identification of proteins in the corona support the notion that its formation may have ambivalent outcomes, since it may contribute to the enrichment of ion corona and at the same time lead to bone cells responses. This bio-complex works as a Trojan horse effect that facilitates nanoparticles internalization since calcium is a key regulator of several cellular functions, while phosphate/amorphous calcium groups can trap several biomolecules. In fact, P and Ca are mineral ions playing a critical role in bone cells biomineralization processes^40^.

The conditions used in the present study have not caused an overt toxicity to bone cells in terms of cell viability, and they did not cause a decrease of total cell numbers in a short term culture model. Human bone cells are slowly proliferating cells, and long-term cultures as well as clonogenic assays could extend the reported results. Electron microscopy has shown indeed cell suffering, a part of which can be reversible under favorable conditions. This may require interruption of cell exposition to titanium nanoparticles, which may not occur *in vivo*, when they are produced by tribocorrosion and continuously released into the surrounding tissue. The presence of autophagolysosomes and its correlation with cell death was already reported in literature^41^. More serious may be the observed damage to the DNA molecules in cells that may proceed to division, since they may lead to mutagenesis (see Fig. 2 of complementary information). Our results are in accordance with literature where it was already reported that TiO_2_ nanoparticles can induce DNA strand breaks and chromosomal damage in bone marrow and/or peripheral blood in mice, and to apoptosis in the mouse liver and spleen^35^,^41^,^42^,^43^. The possible mechanisms for nanoparticles-caused DNA damage may be via oxidative stress, where previous studies showed that TiO_2_ nanoparticles have hydroxyl radical activity triggering reactive oxygen species formation in different cell lines^35^,^41^,^42^,^43^.

We suggest that the bio-complexes internalized by osteoblasts can be dissociated, since no Ca distribution was observed around nanoparticles inside of the cell. Until now, it is unclear how the Ca and P attaching and detaching process occur and how they are influenced by the dynamic biological environment. Further investigations are under way to address these challenges and to potentially explore the mechanism of internalization. The proposed concept of Trojan horse bio-complexes can be used as a step forward for nanoparticle-based approaches for bone applications.

## Material and Method

Titanium dioxide nanoparticles dry powder (Product No. 1317-70-0, particle size <25 nm and spec. surface area: 45–55 m^2^/g) was purchased from Sigma–Aldrich. Dulbecco’s Modified Eagle’s Medium (DMEM) supplemented with 10% (v/v) of fetal bovine serum (FBS, Gibco) was used for osteoblasts culture. Bovine serum albumin (BSA, fraction V, sigma) was used as a stabilizing agent during the optimization protocol of anatase dispersion.

### Physicochemical characterization of anatase nanoparticles

The Brunauer-Emmet-Teller (BET) specific surface area of anatase powder was determined using an (Autosorb-1, Quanta Chrome Instruments). Particle size and morphology of the dry powder and anatase suspension in ultra pure water (condition without dispersion) was taken using a scanning transmission electron microscope STEM (JEOL 2100, 200 kV). For all TEM analyses, a drop of aqueous suspension was placed onto a holey carbon-coated copper grid, air-dried and observed with TEM and STEM. Size distribution of Ti particles was also investigated by dynamic light scattering (DLS) using a ZetaSizer Nano ZS (Malvern Instruments GmbH, Germany). DLS measurements were performed at 25 °C using standard 10 mm disposable optical polystyrene cuvettes. For each condition tested, DLS measurements were performed in triplicate, with the number and duration of sub-measurements for each run determined automatically by the instrument’s software. For all samples, suspension was homogenized by pipetting in and out several times before filling the sample in the cuvette. The phase composition of anatase was determined by X-ray diffraction patterns obtained in the electron microscope (JEOL 2100, 200 kV).

### Ti suspension preparation and characterization

A stock suspension of anatase nanoparticles in ultrapure water at a concentration of 2 mg/mL (pH 4) was prepared. After this procedure, an ultrasonic disintegrator (Q-Sonica 700 W, USA) equipped with a 19 mm Ti tip was used to disperse anatase nanoparticles. Before setup the sonication protocol, a calorimetric method was employed in order to calculate the specific energy as well as the sonication power transferred to the suspension^44^. Sonication was applied until no further advance in the de-agglomeration process was achieved. To reduce an excessive temperature rise, an ice bath used to cool down the suspensions during the direct sonication process. The sonication was then performed at 32 W of delivery acoustic power during 15 min in pulse mode. After 24 h of stabilization, the size distribution of particles was determined by DLS and TEM. The investigation of anatase nanoparticles in cell culture media was carried out by diluting 2 mg/mL anatase suspension in DMEM supplemented with 10% (v/v) FBS, pH 7 to 100 μg/mL DMEM 10% FBS. Due to an increase in nanoparticle aggregation in culture medium, albumin was used as a stabilizer agent. For that, 100 μg/ml of BSA was added to DMEM with 10% FBS. For BSA detection surrounding anatase aggregates, an immunogold labeling was prepared. Samples were never allowed to dry between the different steps, and all the incubations were done at room temperature. Immediately after nanoparticle incubation with culture medium, samples were incubated with a 2% of fish gelatin diluted in PBS. After several washes in ultrapure water, samples were incubated with blocking solution for 30 min. Samples were then washed twice by a 5-min immersion in incubation buffer in PBS. Ten-microliter drops of anti-albumin (Sigma, B1520) diluted 1/1250 in incubation buffer were deposited on grids containing the samples. Antibody incubation was carried out overnight at 4 °C. Samples were then washed in the blocking solution containing fish gelatin 6 times for 5 min in incubation buffer. After washing a secondary antibody with colloidal gold nanoparticles of 10 nm was used. After several washes samples were dried and analyzed by TEM.

### Exploring the nano-bio interface

Anatase suspensions in culture medium supplemented with BSA were investigated by high-resolution transmission electron microscope (HRTEM, JEOL 2100 F operating at 200 kV). Elemental map analyses were also carried out in a JEOL 2100 F equipped with an x-ray Detector (EDX–Energy-dispersive x-ray spectroscopy) in TEM and STEM mode. The crystalline precipitates formed after immersion of anatase in physiological media were characterized by electron diffraction and elemental map analyses were carried out using a Noran Energy EDX spectrometer. For protein adsorption from culture medium to anatase suspensions was investigated by electron energy loss spectrometry (EELS-GIF Tridiem GATAN spectrometer). The samples for TEM observation were prepared by dropping the different concentrations of anatase suspensions (100, 50, 10 and 5 μg/mL) in culture medium with or without SBF onto a holley coated copper grid. The same analyses were performed after 24 h of nanoparticle incubation with culture medium. After naturally drying in air, samples were analyzed. After incubation of anatase nanoparticles with medium culture for 1 hour at 37 °C, samples were ultra-centrifuged at 16000 rpm, during 60 min, at 4 °C. After centrifugation, the pellet was washed 2 times and then re-suspended in 200 μl 1X buffer (Tris-HCL 0,0625 M, SDS 2,5%, 5% 2-Mercaptoetanol, 7% Glicerol (stok) and frozen at −20 °C). Protein adsorption to anatase bio-complex was investigated by SDS-PAGE. Samples were boiled 10 minutes 100 °C and run in 7,5% bis-polyacrylamide gel, 120 V was used to separate proteins by molecular weight. Therefore, gel was stained using colloidal Coomassie blue 1% (BIO-RAD Cat. 161–0406). Protein band were then identified by mass spectrometry.

### Osteoblasts culture

Primary human osteoblasts were isolated from human cancellous bone explants discarded in arthroplasty surgical procedures of adult healthy donors^43^. The consent was obtained from all subjects by the Fluminense Federal University and carried out in agreement with a local Ethic Committee’s register number: #232/08). All experimental protocol was also approved by the same ethic committee. The osteoblast-like cells at 2–6 passages were used for *in vitro* studies.

### High throughput anatase toxicity testing

Cells in log-phase growth were seeded into 96-well microplates (5000 cells per well) and incubated in culture medium for 24 h at 37 °C, 5% CO_2_. After 24 hours, culture medium was removed and replaced with culture medium containing Hoechst 33342. Anatase dispersion was diluted to the different concentrations (100, 50, 10 and 5 μg/mL) and then exposed to osteoblasts culture during 72 hours. The concentrations were special selected by making a literature review with respect to the concentration of particles in the peri-implant environment of patients carrying metallic implants as well as *in vitro* toxicity studies of titanium dioxide debris with focus on implants^3^,^4^,^8^,^45^,^46^,^47^,^48^,^49^,^50^. Images were analyzed directly using the IN Cell Analyzer 1000 Multi Target Analysis Module. Hoechst 33342 was used to identify and segment nuclei as objects. For live and dead assays cells were incubated after 72 hours of exposure to anatase nanoparticles with propidium iodide (10 μg/mL) for 15 minutes. The sample was subsequently read at In Cell Analyzer 2000. Copper oxide with a concentration of 50 μg/ml was used as a positive control. Each experiment was conducted in triplicate.

The cell cycle assay was performed using flow cytometry. Cells were cultured in initial seeded in 6600 cell/cm^2^ during 24 hours. After 24 hours cells were exposed to 5 and 100 μg/ml NPs TiO2 anatase during 72 hours, then washed with PBS buffer, trypsinized and quantified. For cell cycle analyses, 2 × 10^5 ^cell were re-suspended with propidium iodide solution (0.03 mg/mL) and RNase. The DNA content was analyzed by flow cytometry. The dead/apoptotic cells assay, 2 × 10^5 ^cells were analyzed with Dead Cell Apoptosis Kit with Annexin V (life technologies Willow Creek Road Eugene, OR, USA) following the manufacturer instructions. The samples were incubated for 15 minutes with 5 μL of annexin/FITC solution and 0,1 ug of propidium iodide. All analyses were performed in a FACSAria III flow cytometer (DB, Qume Drive San Jose, CA, USA).

### Nanoparticle internalization studies

Cells were plated in a cell density of 40000 cells/cm^2^ and anatase nanoparticles were prepared according to the protocol described above. After 24 hours, cells were exposed to 0 μg/mL (as control), 5 μg/mL and 100 μg/ml of Ti anatase nanoparticles dilutions in culture medium 10% FBS with additional BSA. After 72 hours, cells were trypsinized, washed in PBS and processed for routine TEM. Briefly, cultured cells were centrifuged (2000 rpm, 2 min) after trypsin digestion in culture plates. The cell pellet was fixed in modified Karnovsky’s fixative (2% paraformaldehyde, 2.5% glutaraldehyde in 0.1 M sodium cacodylate buffer, pH 7.2) for 2 h at room temperature. Samples were post-fixed in solution containing 1% osmium tetroxide, 0.8% potassium ferricyanide, and 5 mM calcium chloride and contrasted *in bloc* with 1% of uranyl acetate. Samples were then dehydrated in acetone and embedded in Spurr. Semi-thin sections (3 μm) were stained with toluidine blue and examined under a light microscope to localize cells with visible nucleus. Ultra-thin sections (70 nm) were examined using a Tecnai Spirit G^2^ electron microscope (FEI, Eindhoven, Holland). At least ten cells of each group (control, 5 μg/mL and 100 μg/mL) were analyzed. The sections were also analyzed at the TITAN 80–300 electron microscope (FEI, Netherlands) operating at 300 kV. EDS in Scanning Transmission Electron Microscopy (STEM) mode was applied to investigate regions containing nanoparticles inside of the cells. Specifically, focused probe was raster scanned across the specimen, and at each probe position, the resultant X-ray emission spectrum was recorded. Finally, an elemental map was constructed. For Electron Tomography, thick sections of embedded material were collected and observed in a JEM 2100 microscope operating at 200 kV. Images were recorded in tilt series with 1°-increment intervals using SerialEm software. Tilt series were aligned by cross correlation and tomogram reconstruction calculated by weighted back projection using Etomo program from IMOD software package. Segmentation and 3D model generation were performed using 3dmod program from the same software package.

### Statistical analysis

For high throughput content imaging, each experiment was carried out in triplicates. A response of 100% was considered for the untreated controls (i.e. 100% viability). Means and standard deviations were calculated and statistical significance was evaluated using 2-way ANOVA and Bonferroni posttests. P < 0.0001.

## Additional Information

**How to cite this article**: Ribeiro, A. R. *et al.* Trojan-Like Internalization of Anatase Titanium Dioxide Nanoparticles by Human Osteoblast Cells. *Sci. Rep.* **6**, 23615; doi: 10.1038/srep23615 (2016).

## Supplementary Material

Supplementary Information
