# Supplementary material for: Trojan-Like Internalization of Anatase Titanium Dioxide Nanoparticles by Human Osteoblast Cells
Source: Sci Rep. 2016 Mar 29;6:23615. doi: 10.1038/srep23615 (PMC4810327; doi:10.1038/srep23615)
Supplement: Supplementary Information [file srep23615-s1.pdf]

Trojan-Like Internalization of Anatase Titanium Dioxide Nanoparticles by Human Osteoblast Cells

A. R. Ribeiro[1,2,3*], S. Gemini-Piperni [1,2*], R. Travassos[1], L. Lemgruber[1,4], R. Carvalho[1], A. L. Rossi[5], M. Farina[6], K. Anselme[7], T. Shokhufar[2,8], R. Shahbazian-Yassar[2,9], R. Borojevic[2,10], L. A. Rocha[2,11], J. Werckmann[1,2], J.M. Granjeiro[1, 2,12]

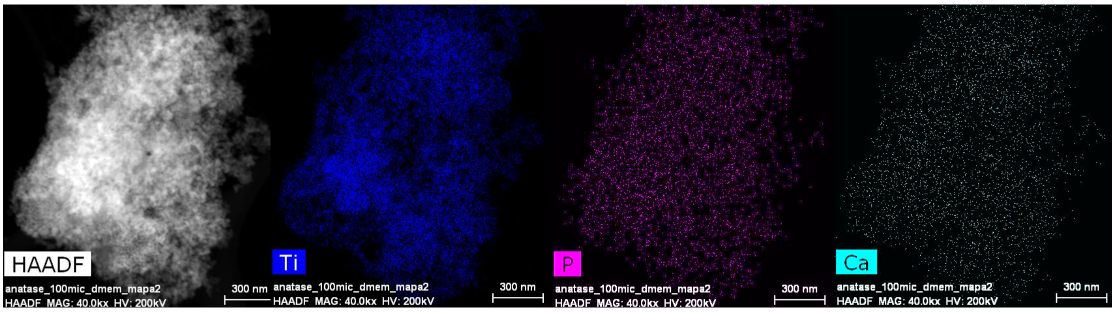

Figure S1: Anatase bio-complexes formation in medium culture without serum and albumin stabilization (100 µg/ml anatase): Dark-field STEM image showing where the corresponding elemental maps were obtained; STEM/EDS Ti-K map; STEM/EDS P-K map, STEM/EDS Ca-K map;

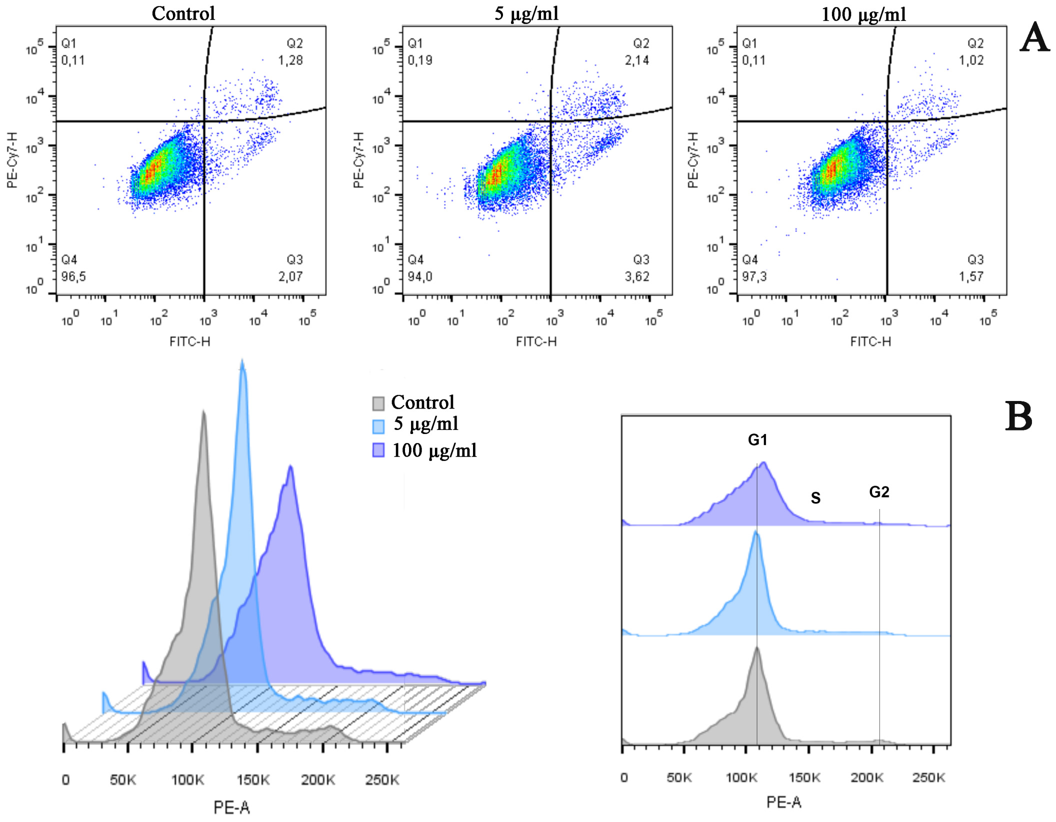

Figure S2: Bone cell viability and cell cycle analysis after exposure to anatase nanoparticles: (A) Dose-dependent effect of anatase on cell apoptosis using flow

cytometry after staining with annexin V / PI; (B) cell cycle analysis.
